# Training Global Leaders in Perioperative Equity: An Evaluation of the CHESA Fellowship

**DOI:** 10.1002/wjs.70481

**Published:** 2026-07-12

**Authors:** Hudson Lin, Rachel Schwartz, Megha R. Aepala, Nour M. Aissaoui, Jacob Serrano, Fred Bulamba, Aleena Jacob, Cathy Kilyewala, Phyllis Kisa, Michael S. Lipnick, Mary T. Nabukenya, Patti Orozco, Doruk Ozgediz, Sriranjani P. Padmanabhan, Cornelius Sendagire, Lia Jacobson

**Affiliations:** ^1^ Center for Health Equity in Surgery and Anesthesia University of California San Francisco California USA; ^2^ School of Medicine University of California San Francisco California USA; ^3^ Department of Anesthesia and Perioperative Care University of California San Francisco California USA; ^4^ Department of Medicine Division of General Internal Medicine University of California San Francisco California USA; ^5^ Philip R. Lee Institute for Health Policy Studies University of California San Francisco California USA; ^6^ University of California Berkeley California USA; ^7^ Elgon Center for Health Research and Innovation Mbale Uganda; ^8^ Department of Surgery Makerere University College of Health Sciences Kampala Uganda; ^9^ Department of Surgery Mulago National Referral Hospital Kampala Uganda; ^10^ Department of Anesthesia Makerere University College of Health Sciences Kampala Uganda; ^11^ Department of Surgery University of California San Francisco California USA; ^12^ Department of Ophthalmology University of California San Francisco California USA; ^13^ Department of Otolaryngology‐Head and Neck Surgery University of California San Francisco California USA

**Keywords:** global health, leadership, mentorship, perioperative equity, program evaluation

## Abstract

**Background:**

Few programs exist to train learners in advancing equity in perioperative care. In response, the UCSF Center for Health Equity in Surgery and Anesthesia (CHESA) launched the CHESA Fellowship in 2021. This 18‐month, non‐ACGME fellowship uses asynchronous modules, expert‐led seminars, mentored research, and community building to equip fellows with the skills to advance perioperative equity worldwide.

**Methods:**

This report describes the fellowship's structure and evaluates its early impact on alumni career development. We conducted a mixed‐methods fellowship evaluation comprising alumni and end‐of‐year surveys, focus groups, and a list of fellow‐authored publications. Surveys assessed skill development, scholarly output, and satisfaction with curriculum elements. Qualitative data from focus groups were coded to highlight fellows' experiences and identify areas for improvement.

**Results:**

Since 2021, CHESA has trained 80 fellows across 18 countries and 12 specialties; 71.3% are from LMICs and 48.8% are female. Surveys demonstrated strong satisfaction with faculty mentorship, project work, and community‐building. Fellows reported acquiring skills in academic writing, mentorship, and leadership, with 53.8% developing new collaborations with co‐fellows and 75.0% integrating fellowship learning into their clinical practice. Among alumni, 76.5% reported advancement into new leadership roles or clinical positions. Across cohorts, alumni published 364 manuscripts during or after the fellowship, with 58.8% focusing on health equity. Fellow and faculty feedback identified needs for enhanced research support and faculty development, which were incorporated into subsequent program iterations.

**Conclusion:**

The CHESA Fellowship offers an innovative model for building global perioperative leadership. The part‐time, virtual format allows fellows to complete the program alongside clinical duties, while seed grants for LMIC fellows support locally driven scholarly projects. By prioritizing cross‐border collaborations between fellows, building long‐term partnerships with LMIC institutions, and ensuring diversity across geography, specialty, and sex, the fellowship balances accessibility with mentorship and community‐building. Alumni report high satisfaction and translate their training into academic, clinical, and advocacy outcomes that advance perioperative equity.

## Graphical abstract

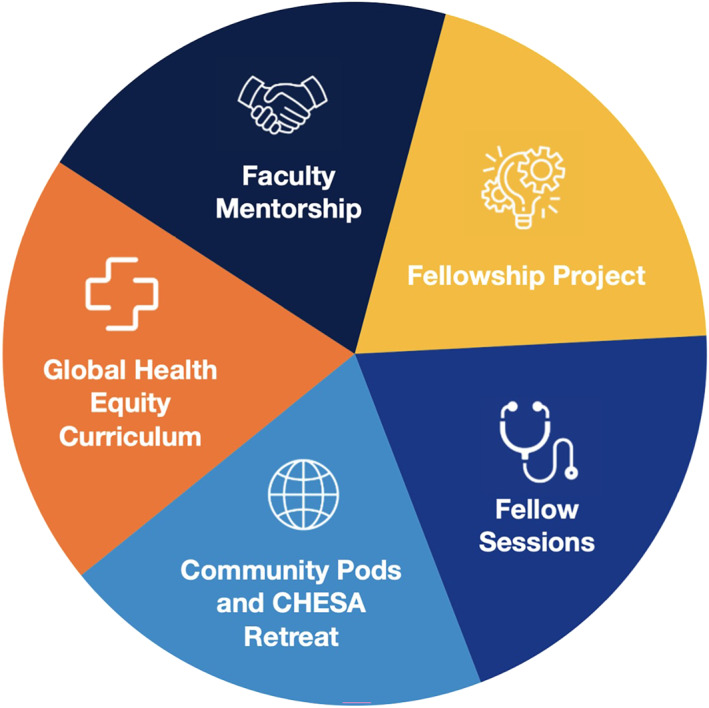

## 1 Introduction

Lack of access to safe, affordable surgical and anesthesia care worldwide accounts for one‐third of global mortality [1, 2]. Low‐ and middle‐income countries (LMICs) face the greatest unmet need, yet receive only 6% of total surgeries performed globally. Impoverishment from medical expenditures is most severe in LMICs, with 81.3 million people driven to catastrophe each year [3]. Even in high‐income countries (HICs), disparities in maternal mortality [4, 5], cancer survival rates [6], and access to analgesia [7, 8] are shaped by race, geography, and socioeconomic status. These findings highlight a critical need to strengthen perioperative capacity in resource‐limited settings.

In the last 2 decades, institutions worldwide have launched global health education initiatives to cultivate leaders in public health. However, these programs have often adopted a narrower scope, focusing on epidemics such as HIV [9, 10] or Ebola [11] or specific regions in Sub‐Saharan Africa [10, 11, 12] or South America [13]. Within perioperative care, collaborative global partnerships have emerged as sustainable strategies to build surgical infrastructure and promote equity [14, 15]. Yet, existing training opportunities in perioperative global health remain scarce, particularly for LMIC trainees [16, 17, 18].

In response, the Center for Health Equity in Surgery and Anesthesia (CHESA) at the University of California, San Francisco (UCSF) launched a Fellowship in 2021 to train future leaders in global surgery and anesthesia. Leveraging a multispecialty leadership team and long‐standing partnerships with LMIC institutions, the fellowship strives to expand equitable access to perioperative care worldwide. This report outlines the fellowship's goals, structure, and impact on alumni careers.

## 2 Methods

### 2.1 Fellowship Description

The CHESA Fellowship is an 18‐month, part‐time, non‐ACGME fellowship that recruits and trains emerging leaders in global surgery, anesthesia, and nursing who use research, education, and advocacy to improve equity in perioperative care. The program is modeled on over 15 years of partnership between UCSF and the Global Partners in Anesthesia and Surgery (GPAS) Senior Scholars program in Uganda, which was created and led by the founding members of CHESA's leadership before CHESA's inception in 2020 [19, 20, 21].

The fellowship operates at no cost to participants. Program activities, including the annual retreat, fellowship projects, and support for a program coordinator, are funded through institutional and grant sources. Faculty contribute their time and mentorship on an in‐kind basis. The core elements of the fellowship are listed below; details on curriculum structure, program funding, selection criteria, and evaluation metrics are included in Supporting Information S1: Materials S1.

#### 2.1.1 Global Health Equity Curriculum

**FIGURE 1:**
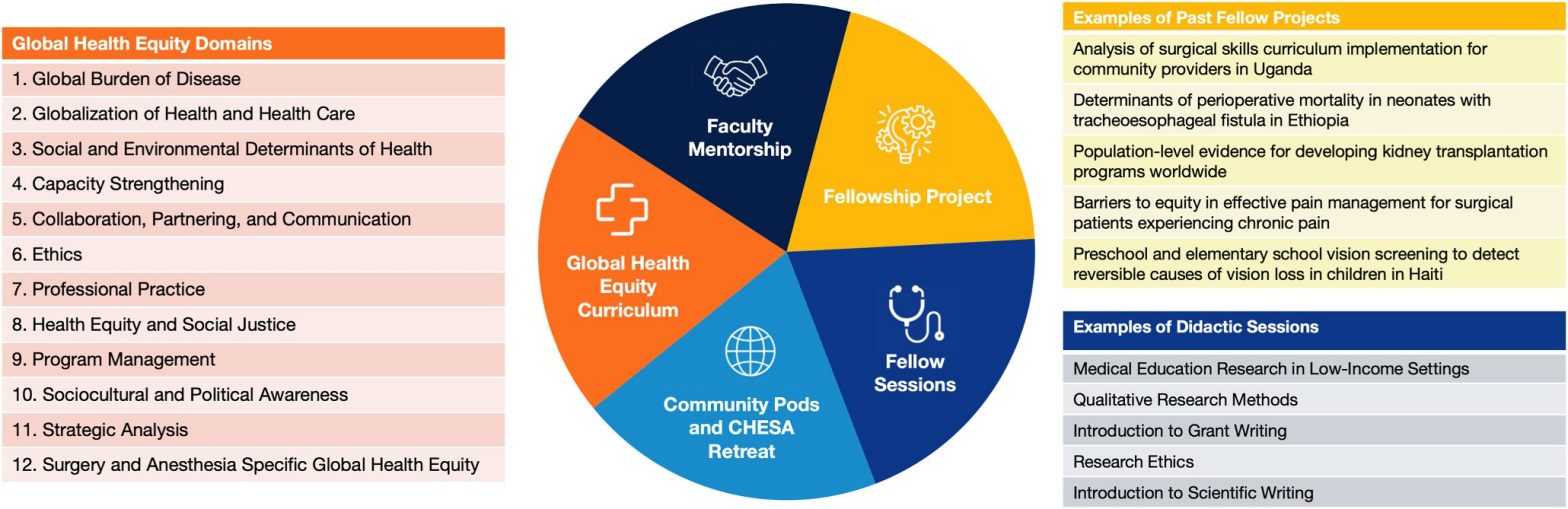
Overview of CHESA fellowship elements.

Fellows complete a self‐guided curriculum, adapted from the Consortium of Universities for Global Health (CUGH) Global Health Competencies Toolkit, including readings and online modules organized by global health equity domain (Figure 1) [22].

#### 2.1.2 Fellow Sessions

Fellows attend virtual sessions, including didactic educational sessions, journal clubs, and Work‐In‐Progress (WIP) meetings. Didactic sessions feature guest speakers covering topics in peri‐operative health equity, professional development, and scientific inquiry, aiming to enhance knowledge and skills in education, research, and advocacy. In journal clubs, fellows lead discussions and critical appraisals of publications addressing perioperative issues globally. WIP meetings allow fellows to present their fellowship projects and garner feedback from CHESA faculty and peers.

#### 2.1.3 Fellowship Project

Central to the CHESA fellowship is a scholarly project aimed at improving training, access, or quality in perioperative care. Fellows apply to the program with a proposed project plan, which is further refined with guidance from a faculty project mentor. The fellowship project is designed to cultivate skills, including project design, scientific writing, and dissemination of findings. Prior fellow projects have targeted curricular reform in surgical training, clinical outcomes research, health systems and policy development, and public health interventions (Figure 1). LMIC fellows are eligible for seed award funding to support project development and dissemination.

#### 2.1.4 Mentorship

Each fellow is paired with specialty‐specific advisors, which include CHESA faculty, fellow alumni, or local mentors at the fellow's home institution. Mentorship is structured around commitments adapted from the AAMC Compact Between Postdoctoral Appointees and their Mentors [23], emphasizing mutual goals around project guidance, feedback, and career development. Mentors meet with fellows at least monthly to provide guidance and monitor progress.

#### 2.1.5 Community Pods and Annual Retreat

Fellows are grouped into Community Pods, consisting of individuals organized by specialty or geography, to facilitate project collaboration and relationship‐building. Pods meet at least every other month and lead one journal club during the fellowship. Each cohort convenes at the CHESA Fellows Retreat, a 3‐day in‐person event with shared learning, community building, and professional and leadership development.

### 2.2 Program Evaluation

The CHESA Fellowship has undergone iterative improvements with each cycle, based on structured feedback from fellows and faculty. This evaluation includes data from fellow and alumni surveys, focus groups, and a fellow publication tracker. This study received exemption from the University of California, San Francisco Institutional Review Board (IRB #24‐42056).

#### 2.2.1 Survey Collection

End‐of‐year surveys from the 2022–23 and 2023‐24 CHESA cohorts assessed the fellowship's impact on participants' skills, career development, and engagement in global perioperative equity efforts. Questions examined satisfaction with curriculum elements and competency in key areas. A 2025 survey was sent to all prior CHESA alumni, focused on scientific productivity since graduation and fellowship impact on career development. Surveys were completed electronically and anonymously to maintain confidentiality and reduce response bias. A representative sample of our end‐of‐year survey is included in Supporting Information S1: Materials S2.

#### 2.2.2 Focus Groups and Qualitative Analysis

Five semi‐structured focus groups were conducted at the 2024 in‐person CHESA Fellows Retreat. Focus groups were led by a member of the CHESA leadership team (D.O., M.S.L., S.P.P., C.K., P.O.) using an interview guide (see Supporting Information S1: Materials S3) developed by the CHESA Fellowship directors (L.J., S.P.P., C.K.). Participants were split into groups of fellows, alumni, or faculty to limit social desirability bias. Sessions ranged from 32 to 52 min and were audio‐recorded and transcribed into Dedoose (version 10.0) for analysis [24]. Focus group analysis was rooted in inductive thematic analysis [25]. R.S. and H.L. developed a set of codes after reviewing each transcript, which was then revised by N.M.A. Two researchers (H.L., N.M.A.) then independently coded transcripts and resolved discrepancies by consensus to create a final codebook. Reflexivity practices are described in Supporting Information S1: Materials S4.

#### 2.2.3 Publication Tracker

We conducted a structured search in June 2025 to evaluate the scholarly output of CHESA fellows during and after their fellowship. Each fellow's name was queried in Google, PubMed, and Google Scholar using combinations of their full name, “publication”, and “UCSF.” Publications were reviewed to identify whether the fellow was listed as first, middle, senior, and/or corresponding author. We also assessed publications for a health equity focus, defined as work that identifies, measures, or addresses health differences across different sociodemographic groups [26, 27, 28]. Two reviewers (MRA, JS) independently screened titles, abstracts, or full texts to classify publications. Fellows were categorized as LMIC or HIC using the 2025 World Bank classification to assess publication trends by income setting.

## 3 Results

Since the Fellowship's inception in 2021, there have been a total of 80 CHESA fellows working in 18 countries (Table 1). Approximately half of CHESA fellows are female (48.8%), and most are early‐career (83.7%) and from an LMIC (71.3%). Twelve specialties are represented, the most common being general surgery (33.7%) and anesthesia (15%).

**TABLE 1:** Summary of CHESA fellows and alumni, 2021–2025.

Variable	Number (%), *n* = 80
Sex	
Male	41 (51.2)
Female	39 (48.8)
Career stage (years post‐training)	
Early‐career (0–4)	67 (83.7)
Mid‐career (5–9)	9 (11.3)
Late‐career (10+)	4 (5.0)
Citizenship	
Low‐ or middle‐income country (LMIC)	57 (71.3)
High‐income country (HIC)	23 (28.7)
Specialty	
General surgery	27 (33.7)
Anesthesia/critical care	12 (15.0)
Otolaryngology	9 (11.3)
Pediatric surgery	8 (10.0)
Urology	6 (7.5)
Clinician researcher	4 (5.0)
Plastic surgery, trauma surgery	3 (3.7)
Ophthalmology, orthopedic surgery, MD student	2 (2.5)
Transplant surgery, neurosurgery	1 (1.3)

### 3.1 Survey Analysis

Eight of 22 fellows (36.4%) completed the 2022‐23 survey. Following its implementation as a graduation requirement in the subsequent cohort, the 2023‐24 survey achieved a 100% completion rate (Table 2). Overall, fellows reported high satisfaction levels with curriculum elements, especially the fellowship project and the opportunity to connect with co‐fellows. 53.8% initiated new academic or clinical collaborations due to their participation in the fellowship, and 91.7% reported feeling more positive about their work as physicians.

**TABLE 2:** Summary of 2023‐24 fellowship end‐of‐year survey.

Question	Category	Avg. rating
How useful was each fellowship program component in helping you achieve your fellowship goals? (1 = not at all useful, 2 = slightly useful, 3 = somewhat useful, 4 = very useful, 5 = extremely useful)	Global health equity curriculum	4.03/5
	Fellow Friday sessions	4.41/5
	Faculty and project mentorship	4.52/5
	Community pods	4.07/5
	Project funding	4.55/5
	Fellowship project	4.69/5
	Global community	4.66/5
Which additional scholarly/educational skills do you want to prioritize for further development? (items ranked from 1‐ most important, to 5‐least important)	Scientific writing	2.48
	Grant writing	2.48
	Curriculum development and evaluation	2.76
	Trainee assessment and feedback	3.52
	Oral presentation skills and slide preparation	3.76

Question	Percent responding “yes”
Were you able to complete your project within the 1‐year timeframe?	23.1%
Have you developed a new friendship with a fellow during the fellowship?	92.3%
Have you developed a new academic/clinical collaboration with a co‐fellow during the fellowship?	53.8%
Have you integrated new approaches/techniques into clinical work due to the fellowship?	75.0%
Have you changed how you serve as a mentor due to the fellowship?	95.2%
Has the fellowship increased how positively you feel about your work as a physician?	91.7%

Notably, only 23.1% of fellows completed their fellowship projects within an academic year. Commonly cited barriers included insufficient time, challenges in obtaining ethics approvals, and conflicts with other professional duties. Iterative improvements made to address the low project completion rate are outlined in the discussion.

The alumni survey achieved a response rate of 61.8% (34 of 55 alumni). Of the respondents, 76.5% attributed promotions and advancements into new leadership roles or clinical positions to their participation in the fellowship. 87.9% believe completing the fellowship supported their ability to publish papers and engage with other public health organizations. 73.5% have presented their CHESA work at a conference, and 58.8% have published their project in a peer‐reviewed journal.

### 3.2 Focus Group Results

Fifteen fellows, seven alumni, and six faculty members participated in the focus groups (Table 3). Analysis of focus groups revealed three core themes: Fellowship Strengths, Fellowship Outcomes, and Areas for Improvement.

**TABLE 3:** Focus group participants.

Focus group	Participant	Sex	Country	Specialty
Faculty group	1	F	HIC	Anesthesia
	2	F	HIC	Orthopedic surgery
	3	M	LMIC	Anesthesia
	4	F	LMIC	Anesthesia
	5	M	LMIC	Pediatric surgery and urology
	6	F	LMIC	Trauma surgery
Fellow group 1	7	M	LMIC	Clinician researcher
	8	M	LMIC	General surgery
	9	M	LMIC	Orthopedic surgery
	10	M	LMIC	Otolaryngology
	11	F	LMIC	Pediatric surgery
Fellow group 2	12	M	LMIC	General surgery
	13	M	LMIC	General surgery
	14	M	LMIC	Otolaryngology
	15	F	LMIC	Otolaryngology
	16	M	LMIC	Pediatric surgery
Fellow group 3	17	F	HIC	Clinician researcher
	18	M	LMIC	Anesthesia
	19	F	LMIC	Orthopedic surgery
	20	F	LMIC	Otolaryngology
	21	F	LMIC	Thoracic surgery
Alumni group	22	F	LMIC	Anesthesia
	23	M	LMIC	General surgery
	24	F	LMIC	Otolaryngology
	25	M	LMIC	Pediatric surgery
	26	M	LMIC	Pediatric surgery
	27	M	LMIC	Pediatric surgery
	28	F	LMIC	Urology

#### 3.2.1 Theme 1: Fellowship Strengths

##### 3.2.1.1 Subtheme 1: Resources and Mentorship

Fellows cited the remote platform as a strength of the fellowship, enabling fellows worldwide to participate and interact on Zoom. They highlighted access to fellowship resources, including online databases, scientific journals, and global equity modules, as valuable assets.

There are so many things you can access and read around…I was actually able to access a grant foundation…and I'm actually applying for one.

CHESA Fellow

Fellows also praised the feedback and support from their faculty mentors, which enabled them to undertake more ambitious projects with regular check‐ins.

Since I became a CHESA fellow, I was exposed to different kinds of mentorship—a mentor that really advanced me in career leadership and a mentor about my research—so I realized that you could not walk alone.

CHESA Fellow

##### 3.2.1.2 Subtheme 2: Collaboration Opportunities

Through the fellowship, trainees gained access to a community dedicated to global surgical equity. Many leveraged shared challenges in perioperative care into opportunities for collaboration:

I've met people doing other projects, and I've actually jumped into their projects… We've met up to really discuss some things, and they can give you insights from different parts of the world.

CHESA Fellow

Within small group pods, fellows praised the flattened hierarchy and peer support.

[A fellow pod member] would send a WhatsApp call like “[NAME], what time can we meet?” So it's like you have people to hold you on either side, and when you're struggling.

CHESA Fellow

#### 3.2.2 Theme 2: Fellowship Impact

##### 3.2.2.1 Subtheme 1: New Skills and Perspectives

Fellows gained competency in research, mentorship, and leadership. Notably, many participants mentioned an increase in research productivity and confidence in generating novel studies:

We have a lot of cases that have not yet been published, especially in the Far East, in the rural areas. But because of CHESA, we are coming up with ideas to start publishing what we are doing so that the world can know….

CHESA Fellow

Participants described CHESA mentors' modeling as shaping their approach to mentoring trainees within their institutions. Many mentioned how the fellowship equipped them with tools to assume new leadership roles, where they hoped to change local policies and drive systemic change.

Overall, fellows described a shift in their perspective on their roles as surgeons and anesthesiologists. Through the global equity curriculum, participants gained insight into the impact of systemic inequities on surgical outcomes and the importance of enhancing surgical safety in their daily practice.

I realized there are quite a number of things, socially, a lot of social injustices, that eventually lead to this person's sickness or their failure to get treatment. And so, if there's anything I learned from the fellowship, it was to advocate for better services for the patient.

CHESA Fellow

#### 3.2.3 Subtheme 2: Future Goals

Looking forward, many fellows hoped to continue their projects and implement findings in local practice. Others described a desire to disseminate learning to peers and encourage them to apply for the fellowship. Both fellows and faculty emphasized the importance of sustained support structures for alumni.

As CHESA alumni, we should create a club where we foster each other’s ideas, hold each other's hands, review each other's research.

CHESA Fellow

#### 3.2.4 Theme 3: Areas for Improvement

One commonly cited infrastructure need was additional understanding regarding the IRB process, acquiring funding, and publishing research. Fellows underscored the importance of early exposure to these trainings so they could begin the fellowship with IRB approval in place.

Funding and skills in knowing the opportunities and channels where people can publish their project. I think this is a very important thing we need.

CHESA Fellow

Similarly, CHESA faculty recommended additional skills training to better support their mentees, with suggested topics including mentorship, project management, and feedback:

I think [a faculty development series] would be fantastic. And then I think that faculty would feel more engaged, and more connected to CHESA, connected to the fellows, connected to the work.

CHESA Faculty

Both fellows and mentors advocated for more in‐person engagement beyond the annual retreat as a path to create and sustain connections and shared work. Others expressed a desire to be exposed to high‐income surgical settings to enrich their understanding of surgical disparities and exposure to the most advanced technologies.

### 3.3 Publications

The June 2025 literature search yielded 364 total publications co‐authored by 44 CHESA alumni during and after their fellowship (Table 4). On average, HIC authors published more manuscripts than LMIC authors (mean 11.7 vs. 5.4) and had a higher proportion of first‐author publications (32.9% vs. 21.5%) but a lower proportion of senior‐author (4.7% vs. 10.0%) or corresponding‐author publications (39.3% vs. 51.5%). Most manuscripts focused on health equity, with a higher proportion among LMIC authors (66.2% vs. 54.7%).

**TABLE 4:** Publications from CHESA alumni during and after fellowship.

Citizenship (*n*)	Publications				
	First author (% of total)^a^	Senior author (% of total)^a^	Corresponding author (% of total)^a^	Health equity focus (% of total)^a^	Total
LMIC (24)	28 (21.5%)	13 (10.0%)	67 (51.5%)	86 (66.2%)	130
HIC (20)	77 (32.9%)	11 (4.7%)	92 (39.3%)	128 (54.7%)	234
Total (44)	105 (28.8%)	24 (6.6%)	159 (43.7%)	214 (58.8%)	364

^a^ Percentages are calculated as a proportion of the total publications within each citizenship group.

## 4 Discussion

Since its launch in 2021, the CHESA Fellowship has fostered a growing network of surgeons and anesthesiologists engaged in health equity research and advocacy. To our knowledge, it is the largest and most geographically diverse perioperative global health fellowship to date.

Published literature on global surgery fellowship programs remains sparse. While broader global health programs share structural elements of mentorship and experiential learning [29], perioperative‐specific programs have either prioritized HIC participants or have launched recently and have yet to publish program details [16]. CHESA fellowship's distinguishing features—including majority LMIC enrollment and seed grant funding—reflect a deliberate effort to fill this gap.

Peer‐led pods and the CHESA retreat foster a robust global community and alumni network, with most fellows developing new academic and clinical collaborations. The virtual format has proven scalable and accessible, allowing the fellowship to expand its cohort while maintaining an almost 3:1 ratio of LMIC‐to‐HIC fellows. Same‐country mentor pairings and long‐term partnerships with LMIC institutions—including Makerere University in Uganda—help fellows feel supported in their home contexts [30, 31]. The locally adaptable curriculum also lowers barriers for LMIC‐led institutions seeking to develop similar programs. Longitudinal mentorship and global engagement with peers can be replicated through existing regional platforms, and where funding is unavailable, fellows can integrate projects into existing institutional roles through quality improvement or education. Already, alumni have begun translating their training into new initiatives, such as a global surgery workshop and mentorship program developed for the College of Surgeons of East, Central, and Southern Africa (COSECSA) [32].

One key challenge identified within the fellowship was primary project completion within a single academic year. We have addressed this by extending the fellowship period to 18 months, rearranging the curriculum to better support early project development, and supplementing the curriculum with tailored research modules. Despite these modifications, external challenges persist, including delays to ethics board approvals, heavy clinical responsibilities, and periods of social or political instability. While acknowledging broader systemic constraints, we approached some of these challenges by leveraging our extensive alumni network to better support fellows, providing frameworks for early ethics review submissions and requesting employer‐protected time for fellow participation.

From a research productivity perspective, CHESA alumni published a mean of 6.5 manuscripts during and after their fellowship in a mean period of 2.7 years. Notably, over half of all fellow publications centered on health equity topics, reflecting a strong alignment between the fellowship's mission and scholarly output. Publication trends between HIC and LMIC fellows were largely consistent with prior literature, with the latter proportionally underrepresented in total output [33]. This disparity may be partially explained by structural inequities in training—many HIC fellows benefit from dedicated, funded research years, whereas LMIC fellows must balance research endeavors with ongoing clinical responsibilities. LMIC authors did achieve a higher rate of corresponding and senior authorship than HIC authors, potentially bolstered by CHESA seed grants and longstanding institutional partnerships that promote professional development for LMIC faculty [34].

### 4.1 Limitations

Our evaluation of the fellowship has several limitations. Despite efforts to standardize publication searches on multiple platforms, we relied on global academic databases, which could overlook publications from non‐indexed journals, particularly by LMIC fellows. Although our analysis measured an increase in fellows' self‐judgment of research competency, we lacked more objective measures of research impact, especially for gauging whether CHESA narrowed any pre‐existing productivity gaps between HIC and LMIC fellows. Research productivity and senior authorship may also increase with career advancement, independent of fellowship participation. Additionally, while end‐of‐year surveys were anonymized to reduce social desirability bias, their completion as a graduation requirement may have introduced pressure to respond favorably. The survey instrument itself was developed specifically for this program and has not undergone formal psychometric validation, which may limit the interpretability of quantitative findings.

### 4.2 Future Directions

Based on fellow and alumni surveys and focus groups, upcoming cohorts will participate in a pre‐fellowship session on IRB processes and receive research support through targeted modules and a database of open‐access resources. We aim to expand clinically‐focused opportunities through international exchanges, fostering bidirectional training and collaboration as the CHESA community grows. Future evaluation will measure long‐term, systems‐level impact, including policy shifts, organizational growth, and improvements in local health outcomes [35]. Sustained alumni engagement—through mentorship of incoming fellows and involvement in CHESA activities—will be essential for tracking these metrics and advancing toward a more equitable global perioperative landscape.

### 4.3 Conclusion

The CHESA Fellowship represents an innovative model for training future leaders in global perioperative equity. Our remote, multidimensional format effectively drives research productivity, mentorship, and cross‐border partnerships while remaining accessible for trainees worldwide. Feedback from focus groups and surveys reveals broad satisfaction with the curriculum, while facilitating iterative improvements for subsequent cohorts. Post‐graduation, alumni remain engaged in health equity projects and the CHESA community, and most report incorporating their training into clinical practice. Future efforts will focus on strengthening research support, expanding clinical exchanges, and sustaining alumni engagement to amplify the fellowship's long‐term impact.

## Author Contributions

**Hudson Lin:** investigation, writing – original draft, formal analysis, data curation, methodology, visualization, validation. **Rachel Schwartz:** conceptualization, formal analysis, investigation, methodology, validation, writing – review and editing. **Megha R. Aepala:** data curation, investigation, writing – review and editing. **Nour M. Aissaoui:** formal analysis, investigation, writing – review and editing. **Jacob Serrano:** data curation, formal analysis, investigation. **Fred Bulamba:** supervision, validation, writing – review and editing. **Aleena Jacob:** data curation, formal analysis, investigation, writing – review and editing. **Cathy Kilyewala:** conceptualization, methodology, project administration, validation, writing – review and editing. **Phyllis Kisa:** supervision, validation, writing – review and editing. **Michael S. Lipnick:** conceptualization, validation, writing – review and editing. **Mary T. Nabukenya:** supervision, validation, writing – review and editing. **Patti Orozco:** data curation, investigation, writing – review and editing. **Doruk Ozgediz:** conceptualization, validation, writing – review and editing. **Sriranjani P. Padmanabhan:** conceptualization, methodology, project administration, validation, writing – review and editing. **Cornelius Sendagire:** supervision, validation, writing – review and editing. **Lia Jacobson:** conceptualization, methodology, project administration, supervision, validation, writing – original draft, writing – review and editing.

## Funding

The authors have nothing to report.

## Consent

Informed consent was obtained from all individual participants included in the study.

## Conflicts of Interest

The authors declare no conflicts of interest.

## Supporting information


Supporting Information S1

## Data Availability

The data that support the findings of this study are available from the corresponding author upon reasonable request.
