# Supplementary material for: Training Global Leaders in Perioperative Equity: An Evaluation of the CHESA Fellowship
Source: World J Surg. 2026 Jul 12;50(8):2329–37. doi: 10.1002/wjs.70481 (PMC13460455; doi:10.1002/wjs.70481)
Supplement: Supplementary file 1 [file WJS-50-2329-s001.docx]

**Supplemental Materials S1: CHESA Fellowship Program Structure and Operations**

2024-2026 Cohort Agenda:

| **Date** | **Topic** | **Global Health Equity Domain** |
| --- | --- | --- |
| 20 Sept 2024 | CHESA Fellowship Orientation |  |
| 7 Oct 2024 | Global Surgery and Anesthesia 101 | Surgery and Anesthesia Specific Global Health Equity Competencies |
| 18 Oct 2024 | Career Planning + Project/Time Management |  |
| 4 Nov 2024 | Research Ethics | Ethics Competencies |
| 15 Nov 2024 | Qualitative Methods |  |
| 2 Dec 2024 | Journal Club 1 | Global Burden of Disease Competencies |
| 20 Dec 2024 | Journal Club 101 + Implementation Science |  |
| 6 Jan 2025 | Quality Improvement |  |
| 17 Jan 2025 | Medical Education Research in Low-Income Settings |  |
| 3 Feb 2025 | Journal Club 2 | Globalization of Health and Health Care Competencies |
| 21 Feb 2025 | Fireside Chat with CHESA and COSECSA Leadership, Interim Fellowship Feedback Session | Collaboration, Partnering, and Communication |
| 3 March 2025 | Optional Admin Office Hours |  |
| 7 April 2025 | Scientific Writing - Session 1 |  |
| 14 April 2025 | Scientific Writing - Session 2 |  |
| 28 April 2025 | Scientific Writing - Session 3 |  |
| 5 May 2025 | Scientific Writing - Session 4 |  |
| 16 May 2025 | Humanitarian Surgery |  |
| 2 June 2025 | Fellow Work in Progress 1 | Professional Practice Competencies |
| 20 June 2025 | Fellow Work in Progress 2 |  |
| 27 June 2025 | General Fellows Work in Progress |  |
| 7 July 2025 | Journal Club 3 | Program Management Competencies |
| 18 July 2025 | Fellow Work in Progress 3 |  |
| 4 August 2025 | Fellow Work in Progress 4 | Strategic Analysis Competencies |
| 15 August 2025 | Fellow Work in Progress 5 |  |
| 19 Sept 2025 | Grant Writing |  |
| 6 Oct 2025 | Grant Writing 2 |  |
| 17 Oct 2025 | How to Publish a Manuscript & Respond to Comments |  |
| 3 Nov 2025 | CHESA Fellows Retreat | Health Equity and Social Justice Competencies |
| 21 Nov 2025 | Journal Club 4 |  |
| 1 Dec 2025 | Fellow Work in Progress 6 |  |
| 5 Jan 2026 | Journal Club 5 | Sociocultural and Political Awareness Competencies |
| 16 Jan 2026 | Fellow Work in Progress 7 |  |
| 2 Feb 2026 | Graduation - End-of-Fellowship Celebration |  |

Self-Led Global Health Curriculum:

| **Global Health Equity (GHE) Competency / Domain** | **Online modules** | **Recommended readings** |
| --- | --- | --- |
| **Domain 1. Global Burden of Disease** | [Global Health 101x online](https://courses.ucsf.edu/course/view.php?id=2489) | [Challenges with DALY Metrics in Surgical Disease](https://drive.google.com/file/d/1UKtVwtVUaXW_A6ruQDR0Dp1jZGYdr01j/view?usp=sharing) |
|  |  | [Key concepts for estimating the burden of surgical diseases](https://drive.google.com/file/d/1bha-Ln8GpjRpxiQQJQs8zsoKIgHRXOSY/view?usp=sharing) |
|  |  | [African Surgical Outcomes Study](https://drive.google.com/file/d/1yJ18G7Q_1-TZ4ZBpnW9zrOP4wOYjm6eN/view?usp=sharing) |
|  |  | [Emergency Abdominal Surgery Mortality by Income Level](https://drive.google.com/file/d/11BqRBYySzJnO-vR6kh8Em3KUWSkypIfG/view?usp=sharing) |
|  |  | [Reassessing Metrics for Surgical Disease: Addressing Ethical Implications of Burden, Need, and Access to Care](https://drive.google.com/file/d/1T7T0QiUBadLF-HPLMIz33IEkDx6hhsGN/view?usp=sharing) |
| **Domain 2. Globalization of Health and Health Care** |  | [Global surgical ecosystems - need for strengthening](https://pubmed.ncbi.nlm.nih.gov/27986227/) |
|  |  | [Primer about health insurance and the uninsured](https://www.kff.org/uninsured/report/the-uninsured-and-the-aca-a-primer-key-facts-about-health-insurance-and-the-uninsured-amidst-changes-to-the-affordable-care-act/) |
|  |  | [10 things to know about Medicaid](https://www.kff.org/medicaid/issue-brief/10-things-to-know-about-medicaid-setting-the-facts-straight/) |
|  |  | [Safety net health systems at risk: who bares the burden of uncompensated care](https://www.healthaffairs.org/do/10.1377/hblog20180503.138516/full/) |
|  |  | [Characteristics of safety net hospitals](https://www.ncbi.nlm.nih.gov/books/NBK401306/) |
|  |  | [Alma-Ata at 40 years: reflections from the Lancet Commission on Investing in Health](https://drive.google.com/file/d/1eZWHrtwENcCXyDiVHDR5IDTgqCISGuoy/view?usp=sharing) |
|  |  | [Health Systems Monitoring: Indicators & Measurement Strategies Handbook](https://drive.google.com/file/d/1-VB2Mlf5C48UoHsk5DFLacQzVklp3hVr/view?usp=sharing) |
| **Domain 3. Social and Environmental Determinants of Health** | [Injection injury in Uganda](https://www.youtube.com/watch?v=-o3-DFniIcU) | [CHESA Surgery in Humanitarian Crises Curriculum](https://docs.google.com/document/d/1mWrQ8h7k9svcqB1zZ3Igatv5ZdiuFWEg-iBmoE3xTm0/edit#heading=h.mewd8laoy6pk) |
|  |  | [Impact of Education Program on Labor Epidural Utilization Among Hispanic Medicaid Beneficiaries: A Randomized Controlled Trial](https://drive.google.com/file/d/1sKdnzSOwuQripuvAIbDsS79WFD5N4kBm/view?usp=sharing) |
|  |  | [Too far to walk: maternal mortality in context](https://pubmed.ncbi.nlm.nih.gov/8042057/) |
| **Domain 4. Capacity Strengthening** | [Global Surgery Framework](https://pubmed.ncbi.nlm.nih.gov/33908733/) | [Cardiac Surgery](https://pubmed.ncbi.nlm.nih.gov/30819574/) |
|  | [Partnership Lessons](https://pubmed.ncbi.nlm.nih.gov/32206345/) | [Hernia task shifting](https://pubmed.ncbi.nlm.nih.gov/33427884/) |
|  | [Training partnerships](https://pubmed.ncbi.nlm.nih.gov/33159525/) | [4 delays of global surgery education](https://www.aasurg.org/blog/run-through-a-troop-leap-over-a-wall-the-4-delay-model-of-access-to-global-surgery-education/) |
|  |  | [Capacity-Building Partnerships for Surgical Post-Graduate Training in LMICs](https://drive.google.com/file/d/1g4XF5IUlNThioueKqgqkM0GSmog2luWW/view?usp=sharing) |
|  |  | [Building and Sustaining a Participatory Partnership for Pediatric Surgery in Tanzania](https://drive.google.com/file/d/1k2Cn8PB1mT814HsdboJlAstxu0VSqKWJ/view?usp=sharing) |
|  |  | [Global Surgery Partnerships: A Systematic Review and Sustainability Framework](https://drive.google.com/file/d/1l1K8hCbA9zki7cp055cHPbsWWTM0vGtA/view?usp=sharing) |
|  |  | [Making Cardiac Surgery Feasible in Africa: Lessons from Namibia, Uganda, and Zambia](https://drive.google.com/file/d/1x4RiUxKB2r7CGF2x-YOONvR023wH39bH/view?usp=sharing) |
|  |  | [The 4-Delay Model for Access to Global Surgery Education](https://drive.google.com/file/d/198CAAKEGKJp1kubtFPOEHx3p5TXo0dwV/view?usp=sharing) |
|  |  | [Outcomes of Elective Inguinal Hernia Repair by Associate Clinicians vs. Doctors in Sierra Leone: A Randomized Trial](https://drive.google.com/file/d/1ZT9lKhl82_UgxEd8Xu6I_cfiSzukh7hR/view?usp=sharing) |
|  |  | [The future of global health education: training for equity in global health](https://drive.google.com/file/d/1GBfQHUXQcyuzdK7KagRgRIkYMM6699qL/view?usp=sharing) |
| **Domain 5. Collaboration, Partnering and Communication** | [UW Pain and Policy Modules](https://drive.google.com/open?id=1N7j8WDEj5GFcwAfvS-xxo1qZkxjR_o0u) | [Host Community Views on Trainees in Short-Term Global Health Programs](https://drive.google.com/file/d/1Qo8WOv649fLMYtOroMJcaw51DNXwWbzQ/view?usp=sharing) |
|  | [Kampala Trauma Course](http://www.kampalatrauma.com/lectures/) | [How not to write about global health](https://gh.bmj.com/content/5/7/e003164) |
|  | [Uncommon Service: rural surgical innovation](https://www.youtube.com/watch?v=6upVQu_a0oM) | [Lessons from a partnership in surgical care](https://gh.bmj.com/content/5/3/e002118) |
|  | [Aravind Eye Hospital](https://www.youtube.com/watch?v=MhhvNLztZKI&t=534s) | [Nine Problems That Hinder Partnerships in Africa](https://drive.google.com/file/d/1_ve4RaCf0Sz5lo9xhydTMdMPh9GNCB7W/view?usp=sharing) |
|  |  | [Host community perspectives on trainees participating in short-term](https://www.ncbi.nlm.nih.gov/pubmed/27762010) |
|  |  | [Elevator Pitch: Essential Tips for Effective Communication](https://drive.google.com/file/d/1IP1BAZS6UpBuFuUGeY2dJAcyag-TLT2d/view?usp=sharing) |
| **Domain 6. Ethics** | [Global Health Ethics Course](http://ethicsandglobalhealth.org/) | [Sevens sins of humanitarian medicine](https://drive.google.com/file/d/1wLLBp2L6rK9A-E_K81t6rYgiioi5xIl2/view?usp=sharing) |
|  |  | [CHESA Reading List - Global Health Equity](https://docs.google.com/spreadsheets/d/1wvwrbLyrI0e_foFlSP1hAKR45pJxa0JC2MVLWlAD1dM/edit#gid=891834841) |
|  |  | [The future of global health education: training for equity](https://www.ncbi.nlm.nih.gov/pmc/articles/PMC5117699/) |
|  |  | [Alma-Ata at 40 years: reflections from the Lancet Commission on Investing in Health.](https://www.ncbi.nlm.nih.gov/pubmed/30343859) |
|  |  | [Ethical Issues in Patient Safety Research: Interpreting Existing Guidelines](https://drive.google.com/file/d/10IjZqauJBh59N1BcieubR6WRDzWgk2zV/view?usp=sharing) |
| **Domain 7. Professional Practice** |  | [Surgical Care at the District Hospital WHO](https://apps.who.int/iris/handle/10665/42564) |
|  |  | [Surgical Innovation in LMIC](https://www.sciencedirect.com/science/article/abs/pii/S1479666X21001852) |
|  |  | [Anesthesia at the district hospital WHO](https://apps.who.int/iris/handle/10665/42193) |
| **Domain 8. Health Equity and Social Justice** | [Systemic racism module](https://docs.google.com/presentation/d/1-g6d1WG50yiaBuzg5GOb-ZUbhLtXvJ-yv0jhy6PYO7A/edit?usp=sharing) | [CHESA Reading List - Structural Violence, disparities in periop care](https://docs.google.com/spreadsheets/d/1wvwrbLyrI0e_foFlSP1hAKR45pJxa0JC2MVLWlAD1dM/edit#gid=891834841) |
|  |  | [How childhood trauma affects across a lifetime](https://www.youtube.com/watch?v=95ovIJ3dsNk) |
|  |  | [Black children more likely to die after surgery](https://www.nytimes.com/2020/07/20/health/black-children-surgery-deaths.html) |
|  |  | [Racial bias in pain assessment and treatment](https://pubmed.ncbi.nlm.nih.gov/27044069/) |
|  |  | [US surgeons perceptions of racial ethnic disparities in healthcare](https://pubmed.ncbi.nlm.nih.gov/26818695/) |
|  |  | [Adultification of black children in pediatric anesthesia](https://pubmed.ncbi.nlm.nih.gov/31295177/) |
|  |  | [RCT Education intervention impact on epidural utilization on Hispanic Medicaid beneficiaries](https://pubmed.ncbi.nlm.nih.gov/31299658/) |
| **Domain 9. Program Management** |  | [Rapid and rigorous impact evaluation](https://www.cgdev.org/sites/default/files/Rapid-evaluation-background-paper.pdf) |
|  |  | [Ten reasons not to measure impact](https://ssir.org/articles/entry/ten_reasons_not_to_measure_impact_and_what_to_do_instead) |
|  |  | [The Improvement Guide: A Practical Approach to Enhancing Organizational Performance](https://www.ihi.org/resources/Pages/Publications/ImprovementGuidePracticalApproachEnhancingOrganizationalPerformance.aspx) |
| **Domain 10. Sociocultural and Political Awareness** |  | [Principles of Environmentally Sustainable Anesthesia: A Global Consensus Statement from the World Federation of Societies of Anaesthesiologists](https://drive.google.com/file/d/12MI6Xk6lB2sbOzDVBsfB5uMKWUNrPUUQ/view?usp=sharing) |
|  |  | [Contextual Challenges to Safe Surgery in a Resource-limited Setting](https://drive.google.com/file/d/1R4JBBTDT8Nro0uQIZQ8iarLCL-VYSY-7/view?usp=sharing) |
|  |  | [Defining Global Surgery: The Multidisciplinary Interface of Surgery, Anesthesia, and Public Health](https://drive.google.com/file/d/1kZxrLsMHCX02qfTRKzAXidb4JHvzq537/view?usp=sharing) |
| **Domain 11. Strategic Analysis** |  | [The path to safe and accessible anaesthesia care](https://www.ijaweb.org/article.asp?issn=0019-5049;year=2019;volume=63;issue=12;spage=965;epage=971;aulast=Law) |
|  |  | [Example of Community Needs Assessment: A comprehensive, multitiered, targeted community needs assessment model: methodology, dissemination, and implementation](https://drive.google.com/file/d/13iLBFnbSJN8Su1rnB4l-zuSbmMHUPoJJ/view?usp=sharing) |
|  |  | [Context-Specific Interventions: The impact of trauma care systems in low-and-middle income countries](https://www.annualreviews.org/doi/10.1146/annurev-publhealth-032315-021412?url_ver=Z39.88-2003&rfr_id=ori%3Arid%3Acrossref.org&rfr_dat=cr_pub++0pubmed) |
|  |  | [Use of the six core surgical indicators from the Lancet Commission on Global Surgery in Colombia: a situational analysis](https://www.thelancet.com/journals/langlo/article/PIIS2214-109X(20)30090-5/fulltext) |
|  |  | [Situational Analysis of Essential Surgical Care Management in Iran Using the WHO Tool](https://www.ncbi.nlm.nih.gov/pmc/articles/PMC4939228/) |
| **Domain 12. Surgery and Anesthesia Specific Global Health Equity** |  | [Dare et al 2014 Global surgery: defining an emerging global health field](https://pubmed.ncbi.nlm.nih.gov/24853601/) |
|  |  | [Bath et al 2019 What is ‘global surgery’? Defining the multidisciplinary interface between surgery, anaesthesia and public health](https://gh.bmj.com/content/bmjgh/4/5/e001808.full.pdf) |
|  |  | [Alayande et al 2022 Shifting global surgery's center of gravity](https://pubmed.ncbi.nlm.nih.gov/35715233/) |
|  |  | [NPR Goats & Soda 2019 "Why Surgeons are Rethinking the Fly-In Medical Missions" (on last slide)](https://www.npr.org/sections/goatsandsoda/2019/03/27/656172038/is-it-time-to-rethink-the-fly-in-medical-mission) |
|  |  | [Contextual Challenges in Global Surgery](https://journals.lww.com/annalsofsurgery/fulltext/2018/03000/contextual_challenges_to_safe_surgery_in_a.13.aspx) |
|  |  | [Sustainable Standards in Anesthesia](https://pubmed.ncbi.nlm.nih.gov/34724710/) |
|  |  | [LCOGS 2015 Lancet](https://www.thelancet.com/pdfs/journals/lancet/PIIS0140-6736(15)60160-X.pdf) |
|  |  | [LCOPP 2017 Report](https://www.thelancet.com/commissions/palliative-care) |
|  |  | [UHC, intersectoral action for health - key messages from DCP](https://pubmed.ncbi.nlm.nih.gov/29179954/) |
|  |  | [Essential surgery, key messages from DCP3](https://pubmed.ncbi.nlm.nih.gov/25662414/) |
|  |  | [Cost-effectiveness of surgery and its policy implications](https://www.thelancet.com/journals/langlo/article/PIIS2214-109X(14)70213-X/fulltext) |
|  |  | [A cost effective small hospital in Bangladesh](https://www.ncbi.nlm.nih.gov/pubmed/12676406) |
|  |  | [Health equity in periop care](https://pubs.asahq.org/monitor/article/85/S10/45/117597/What-Is-the-Role-for-Anesthesiologists-and#.YVeMfjYTIkJ.twitter) |
|  |  | [Primer on common perceptions of US role in ‘global health’](https://www.kff.org/global-health-policy/report/the-u-s-government-engagement-in-global-health-a-primer/) |
|  |  | [Joint Statement on Controlled Medicines In Emergencies](https://www.incb.org/incb/en/news/news_2021/incb--unodc-and-who-joint-statement-on-access-to-controlled-medicines-in-emergencies.html) |

Funding Details:

The fellowship is primarily supported by private institutional and individual donor funding, supplemented by internal institutional funding from multiple UCSF departments. This covers program coordination, fellow project funding, professional development support, and one in-person convening per cohort. LMIC fellows without access to external funding at their home institutions may receive up to $8,000 in project seed funding and up to $3,000 for professional development and travel support for conferences. Faculty engagement, including program co-directors and faculty, remains largely voluntary.

Selection Criteria:

Applications are reviewed using a structured scoring rubric across two sections, each worth 50% of the total score. Section 1 evaluates the applicant's background, short answer responses, and video essay, assessing communication skills, prior qualifications, and potential for growth through the fellowship. Section 2 evaluates the proposed project on feasibility, mission alignment, patient-level impact, and overall strength for a candidate of that training level. Applicants from LMIC settings may also submit a budget form to be considered for seed funding. Scores are reviewed by the fellowship leadership team to make final admissions decisions.

Evaluation Metrics:

Fellow engagement is tracked through attendance at all virtual sessions. A minimum of 75% attendance, or completion of make-up written assignments, is required for fellowship completion. Project progress is overseen by primary mentors and reported to the cohort through Work-in-Progress (WIP) presentations. At the end of the fellowship year, fellows submit a final project report reviewed by the fellowship directors prior to certifying program completion.

**Supplemental Materials S2: CHESA Fellowship 2023-24 Cohort End-of-Year Survey**

1. Years of clinical experience after medical school: _________

2. Where are you based geographically?

o East Africa

o US

o All other regions

3. **Rate the following fellowship program components with respect to how useful they were in helping you achieve your fellowship goals.** (1 = Not at all Useful, 2 = Slightly Useful, 3 = Somewhat Useful, 4 = Very Useful, 5 = Extremely Useful)

- Self-guided global health equity curriculum
- Fellow Friday sessions (including WIPs and Journal Clubs)
- Faculty and project mentorship
- Community pods
- Project funding support for applicable fellows from and based in an LMIC during the fellowship
- Fellowship project
- Global community

4. **Self-guided global health equity curriculum:** Please indicate your level of agreement with the following statements about the self-guided global health equity curriculum. (1 = Strongly Disagree, 2 = Disagree, 3 = Neutral, 4 = Agree, 5 = Strongly Agree)

- The curriculum was clear and comprehensive.
- The curriculum covered topics that are relevant to my field.
- The learning materials were well-organized and easy to follow.
- The curriculum enhanced my understanding of global health equity.
- The assignments and activities were engaging and informative.
- The time required to complete the curriculum was reasonable.
- The curriculum was challenging yet achievable.
- The curriculum helped me develop practical skills applicable to my work.
- The resources provided in the curriculum were of high quality.
- The curriculum effectively integrated theoretical knowledge with practical applications.
- The curriculum met my expectations.
- I feel more prepared to address global health equity issues after completing the curriculum.
- The curriculum provided opportunities for reflection and critical thinking.
- The curriculum was a valuable addition to my fellowship experience.
- I would recommend this curriculum to future fellows.

5. What aspects of the curriculum did you find most beneficial?

6. What improvements would you suggest for the curriculum?

7. **Faculty and Project Mentorship:** How often did you meet with your mentor?

o Less than once a month

o 1-3 times per month

o 4 times per month or more

o Varied (please explain) _____________________

8. Did you feel you received adequate guidance and support from your primary mentor and the CHESA Fellowship to design and implement your project?

o No

o Yes

9. **Community pods:** How often did your pods meet?

o Once a month

o Less than once a month

10. What worked well in your pod?

11. What could have been improved in your pod?

12. Were you able to complete your project within the one-year timeframe? By project completion, we mean implementation of your intervention (if applicable, completing data collection and analysis, and documentation of the final findings in a written report.

o No

o Yes

13. If you were unable to complete your project, why? Check all that apply.

▢ Insufficient time to complete the project within the one-year timeframe

▢ Unexpected personal or family obligations

▢ Health issues

▢ Lack of access to necessary resources or materials

▢ Challenges in obtaining required approvals

▢ Difficulty in coordinating with project partners or stakeholders

▢ Changes in project scope or objectives

▢ Inadequate mentorship or guidance

▢ Conflicts with other professional responsibilities or duties

▢ Financial constraints or funding issues

▢ Limited access to necessary data or information

▢ Unforeseen logistical or operational challenges

▢ Changes in institutional or organizational priorities

▢ Other - please explain ___________________________________

14. **Global Community:** Please indicate your level of agreement with the following statements about your experience being part of the CHESA Fellow community. (1 = Strongly Disagree, 2 = Disagree, 3 = Neutral, 4 = Agree, 5 = Strongly Agree)

- I felt welcomed and included in the CHESA Fellow community.
- I was able to build meaningful professional relationships with CHESA fellows.
- I was able to build meaningful professional relationships with CHESA faculty.
- The networking opportunities within the community were beneficial.
- The community facilitated valuable knowledge and skill sharing.
- I had ample opportunities to engage with my peers in the community.
- The support from the community enhanced my overall fellowship experience.
- I felt comfortable seeking advice and feedback from other fellows.
- Being part of the CHESA Fellow community positively impacted my professional development.
- I feel connected to the wider CHESA community.

15. In the course of your fellowship, have you developed a friendship with at least one fellow you didn’t know before?

o No

o Yes

16. Have you developed a new academic or clinical collaboration with a fellow you didn’t know before?

o No

o Yes

17. Please rate your level of confidence in the following areas related to research, scholarly activities, and educational skills. (1 = Not Confident at All, 2 = Slightly Confident, 3 = Moderately Confident, 4 = Confident, 5 = Very Confident)

- Hypothesis generation / Developing good scientific questions for investigation
- Designing/conducting needs assessments and/or survey research
- Designing/conducting clinical trials
- Biostatistics / statistical analysis for clinical research
- Research project management (budgeting, hiring and supervising research personnel, time management)
- Curriculum development and evaluation
- Trainee assessment and feedback
- Grant writing
- Scientific writing (manuscript and abstract preparation)
- Oral presentation skills and slide preparation

18. Please list all the conferences you attended over the course of the fellowship. If none, indicate N/A or not applicable.

19. Please list any posters, presentations, and publications that you worked on over the course of the fellowship. If none, indicate N/A or not applicable.

20. **Advocacy and Leadership Skills:** Please rate your level of confidence in the following areas. (1 = Not Confident at All, 2 = Slightly Confident, 3 = Moderately Confident, 4 = Confident, 5 = Very Confident)

- Advocating for global health equity policies and initiatives
- Leading multidisciplinary teams in a healthcare setting
- Communicating effectively with stakeholders and policymakers
- Mobilizing resources for global health projects
- Influencing health policy at local, national, or international levels
- Building and sustaining partnerships with other organizations
- Navigating cultural and political dynamics in global health contexts
- Developing strategic plans for global health interventions
- Mentoring and inspiring others in the field of global health
- Negotiating and resolving conflicts in a professional setting
- Public speaking and presenting on global health issues
- Driving change and innovation in healthcare practices
- Promoting sustainable and ethical practices in global health
- Engaging with communities to understand their health needs and priorities
- Utilizing social media and other platforms for health advocacy

21. **Patient care:** Have you integrated any new approaches or techniques into your clinical work as a result of your participation in the fellowship program?

o No

o Yes

22. Prior to the CHESA fellowship, how positively did you feel about your work as a physician?

o Extremely negative

o Somewhat negative

o Neither positive nor negative

o Somewhat positive

o Extremely positive

23. After completing the CHESA fellowship, how positively do you feel about your work as a physician now?

o Extremely negative

o Somewhat negative

o Neither positive nor negative

o Somewhat positive

o Extremely positive

24. Has your involvement with CHESA changed the way you serve as a mentor?

o No

o Yes

25. Please rate your level of confidence in your ability to mentor others in the following topics. (1 = Not Confident at All, 2 = Slightly Confident, 3 = Moderately Confident, 4 = Confident, 5 = Very Confident)

- Career and professional development (ex. mentorship regarding career trajectory, promotion, networking, work-life balance)
- Research or project guidance (examples include scholarly work, abstracts, presentations)
- Clinical mentorship (mentorship in the provision of clinical or surgical care, didactic or otherwise)

26. In what ways have you supported your mentees' growth? Type N/A if you do not have any mentees.

**Supplemental Materials S3: Focus Group Guide**

| **Audience** | **Topic** | **Questions** |
| --- | --- | --- |
| Current Fellows and Alumni | Skills Reflection  (Alumni Only) | What skills or experience did you gain that you have found useful post-fellowship?  In hindsight, what skills or training would have been more useful to you now? What did you learn that you didn’t know you should know? |
|  | Research Skill Needs | What training or skills are you missing to move forward in your CHESA Fellowship projects?  What specific research skills do you feel are essential for your career development? |
|  | Program Highlights and Challenges | What aspects of the program have you found most beneficial or rewarding?  What areas for improvement do you suggest for the program? |
|  | Integration of Skills into Clinical Practice | How have the research, education, and advocacy skills learned in the program translated into your clinical practice? |
|  | Future Engagement and Support | What do you wish you knew about the program before you applied for/started the fellowship?  In what ways do you envision continuing your involvement with the fellowship program post-completion?  How can UCSF CHESA best facilitate continued engagement and support for alumni? |
| Faculty | Areas of improvement | Are there areas of the fellowship program that could be strengthened?  Do you have any suggestions for additional resources or training opportunities to enhance the mentorship experience for mentors and mentees? |
|  | Future Engagement and Support | How do you envision continuing your involvement with the fellowship program?  Are there specific ways you would like to further contribute to the program's objectives? |

**Supplemental Materials S4: Statement on Reflexivity in Qualitative Research**

The coding team consisted of a male third-year medical student and a female second-year medical student (HL, NMA). They maintained reflexivity by acknowledging and discussing their own assumptions throughout the coding process, recognizing how their perspectives could shape data interpretation. After developing an initial draft of themes, the coders consulted with fellowship program leadership (RS, AJ, PO, DO, MSL, CK, SPP, LJ) to integrate additional perspectives from anesthesiologists, surgeons, and health services researchers.
